# An Exploratory Retrospective Analysis of Racial Disparities in Fall-Related Injuries Among Black and White Breast Cancer Survivors Receiving Chemotherapy

**DOI:** 10.3390/ijerph22071129

**Published:** 2025-07-17

**Authors:** Asmaa Namoos, Dina Ramadan, Rashema Meekins, Vanessa Sheppard, Nicholas Thomson

**Affiliations:** 1 Department of Surgery, School of Medicine, Virginia Commonwealth University, Richmond, VA 23284, USA; rashema.meekins@vcuhealth.org (R.M.); nicholas.thomson@vcuhealth.org (N.T.); 2 C. Kenneth and Dianne Wright Center for Clinical and Translational Research, Virginia Commonwealth University, Richmond, VA 23284, USA; dwr94@hotmail.com; 3 Social and Behavioral Sciences-School of Public Health, Virginia Commonwealth University, Richmond, VA 23284, USA; vanessa.sheppard@vcuhealth.org

**Keywords:** breast cancer, chemotherapy, racial disparities, survivorship, TriNetX

## Abstract

Purpose: This exploratory retrospective analysis examined racial disparities in fall-related injuries among Black and White breast cancer survivors who received chemotherapy, focusing on the risks associated with specific chemotherapy regimens. Methods: Using real-world data from the TriNetX research platform, we analyzed a cohort of 3223 Stage I–III breast cancer survivors with complete data on race, chemotherapy exposure, and fall-related injuries. The final sample included only Black and White patients treated with chemotherapy between 1 January 2019 and 31 December 2023. Fall events within six months post-chemotherapy were analyzed. Logistic regression models evaluated associations between chemotherapy type and fall risk by race. Results: Black breast cancer survivors experienced a significantly higher rate of fall-related injuries (14.7%) compared to White survivors (10.0%) (*p* < 0.001). The risk was especially elevated among Black patients receiving Cyclophosphamide, Docetaxel, and Carboplatin. Conclusion: This study highlights racial differences in chemotherapy-associated fall risk. While the findings are observational and limited by data availability, they underscore the need for more inclusive survivorship care and further investigation using detailed clinical and contextual variables. Real-world platforms like TriNetX can help identify early signals of disparities that merit prospective study.

## 1. Introduction

Breast cancer survival rates have seen a remarkable increase over the past few decades, largely due to advancements in early detection, targeted therapies, and chemotherapy [1,2]. This progress has transformed breast cancer from a once often fatal disease to one where long-term survivorship is increasingly common [3]. However, the journey to survivorship is not easy for any woman and is often filled with mental and physical challenges [4]. These challenges can be due to medications taken during the diagnosis and afterward, such as chemotherapy and adjunct hormonal therapy [5,6,7]. Chemotherapy remains a cornerstone in the treatment of breast cancer, offering potent efficacy against tumor cells [8]. Medications such as Doxorubicin, Cyclophosphamide, Paclitaxel, Docetaxel, and Carboplatin are widely used for their ability to improve survival rates [9]. However, these treatments are not without their challenges. The potent effects of these drugs are accompanied by a range of side effects, including neuropathy, muscle weakness, and fatigue, which can significantly increase the risk of falls—a serious and often overlooked complication among breast cancer survivors [10,11]. Racial differences in chemotherapy effects may be influenced by variations in drug metabolism, genetic polymorphisms affecting enzyme activity, and differences in immune responses, which can impact both treatment efficacy and toxicity [12].

Moreover, health disparities can profoundly impact the long-term outcomes of breast cancer survivors and decrease their quality of life [13]. Black breast cancer survivors, for instance, face disproportionate challenges compared to their White counterparts, including limited access to quality care and the financial burden of treatment [14,15]. These inequities lead to varied health outcomes, making racial and ethnic minority females more vulnerable to complications [16]. The choice of chemotherapy, in particular, is sometimes influenced by a patient’s socioeconomic status, depending on the availability and type of health insurance [17]. Some chemotherapy drugs, known for their strong effects, such as dizziness and muscle weakness, increase the likelihood of falls and related injuries among these women [11,18].

This exploratory retrospective study aims to examine disparities specifically between Black and White breast cancer survivors regarding fall related injuries following chemotherapy treatment. By leveraging real-world electronic health records available through the TriNetX research platform, a resource frequently used in oncology and disparities research [19,20,21], we investigate differences in fall risk associated with chemotherapy regimens such as Doxorubicin, Cyclophosphamide, Paclitaxel, Docetaxel, and Carboplatin. Clarifying these disparities may help identify gaps in survivorship care and inform targeted interventions to support equity in cancer care outcomes.

## 2. Methods

This retrospective cohort study included 3223 female breast cancer survivors (Stage I–III), aged 18 years and older. Participants were categorized into two groups for initial descriptive purposes: (1) breast cancer survivors who received chemotherapy, (2) those who experienced fall-related injuries after chemotherapy.

The inclusion criteria required patients aged 18 to 89 years who had undergone treatment with one or more of the following chemotherapy agents: Doxorubicin, Cyclophosphamide, Paclitaxel, Docetaxel, or Carboplatin. Fall-related injuries were considered “linked to chemotherapy” if they occurred within 0 to 6 months after the final recorded chemotherapy administration. This time window was chosen based on existing literature identifying heightened fall risk due to acute chemotherapy-related side effects such as neuropathy and fatigue. The study period spanned from 1 January 2019 to 31 December 2023.

Chemotherapy exposure was defined as receipt of one or more of the specified agents (Doxorubicin, Cyclophosphamide, Paclitaxel, Docetaxel, or Carboplatin) at any point during the treatment course. For descriptive analyses, each patient was counted for every agent received, meaning patients who underwent combination regimens are represented in the percentages for all agents they received. As a result, the reported percentages for each racial group are not mutually exclusive and may total more than 100 percent. The percentages presented for chemotherapy exposure represent the proportion of patients within each racial group who received each agent, with patients included in multiple categories if they received more than one drug.

Data for this study were obtained through Virginia Commonwealth University (VCU)-Health Care Organization (HCO) within the TriNetX research network, which provides access to real-world electronic health record data from a diverse range of healthcare institutions, including medical centers. Of the 11,400 patients initially identified, 3223 met inclusion criteria for the final analysis. Patients were excluded if they had missing data on race, chemotherapy treatment, or fall-related injury status. The VCU HCO dataset within TriNetX allowed for a comprehensive analysis of treatment patterns, fall-related injuries, and racial disparities among breast cancer survivors.

TriNetX is a global health research network that connects healthcare organizations and life sciences companies, utilizing real-world data to accelerate clinical research and the development of new therapies [22]. Through its platform, TriNetX provides access to aggregated and anonymized electronic health record (EHR) data, enabling researchers to conduct observational studies, optimize clinical trials, and generate real-world evidence for healthcare advancements.

One of the primary goals of TriNetX is to improve the diversity of clinical trials, allowing for more representative populations in therapeutic research. The network supports the evaluation of treatment patterns, health outcomes, and disparities across different patient populations, offering valuable insights into racial and socioeconomic factors in disease progression and treatment response.

TriNetX has been widely utilized in medical research to investigate diverse clinical outcomes, including oncology, neurological disorders, cardiovascular health, and geriatric medicine [21,23]. By integrating real-world evidence from a diverse range of healthcare institutions, TriNetX enhances the ability of researchers to identify critical health disparities, optimize patient care, and drive data-driven decision-making in clinical and public health research

International Classification of Diseases (ICD-10) codes were used to accurately identify breast cancer diagnoses and fall-related injuries [24]. These codes provide a standardized method for classifying health conditions, ensuring consistency in data collection and analysis. Breast cancer survivors were identified using ICD-10 code Z85.3, which indicates a personal history of malignant neoplasm of the breast. This code was selected to focus specifically on individuals in the survivorship phase, rather than those undergoing active treatment. Fall-related injuries were identified using codes in the W00–W19 range, which captures various types of falls, including those resulting from slipping, tripping, or falling from furniture (see Table 1). This approach allowed us to assess fall risk during the post-treatment period following chemotherapy.

The Current Procedural Terminology (CPT) codes used to document the administration of chemotherapy drugs in the study. These codes provide a standardized way of identifying and billing for specific chemotherapy treatments administered to breast cancer survivors. Each drug listed is commonly used in the treatment of breast cancer, and its corresponding CPT code is used to track its administration in clinical settings (Table 2). Information on the number of chemotherapy cycles or cumulative dosage was not available in the TriNetX dataset and could not be included in the analysis.

For missing data, the TriNetX platform aggregates real-world data from electronic health records (EHRs) across healthcare organizations, ensuring that all necessary patient data are complete and de-identified. TriNetX’s federated data network maintains rigorous data governance protocols, minimizing the occurrence of missing data. Therefore, for this analysis, there were no missing data for key variables such as diagnosis codes, demographic characteristics, or clinical outcomes.

Statistical Analysis. Our analysis was conducted on the TriNetX platform. Descriptive statistics were used to summarize the demographic characteristics of the study population. Continuous variables, such as age, were presented as means with standard deviations (mean ± SD), while categorical variables, including race and ethnicity, were reported as frequencies and percentages.

Univariate analyses were performed using Chi-square tests for categorical variables and T-tests for continuous variables. Logistic regression analyses were conducted to assess the associations between chemotherapy types, race, and the likelihood of fall-related injuries. Logistic regression models were used to examine the association between chemotherapy type and fall-related injury risk, stratified by race. Odds ratios (OR) and 95% confidence intervals (CI) were calculated for each chemotherapy agent to compare fall risk between Black and White breast cancer survivors.

Chemotherapy assignment was presented descriptively by race. Statistical comparisons were not conducted, as the dataset lacked critical clinical decision-making variables (e.g., staging, tumor biology, provider rationale) necessary to contextualize observed differences.

Software and Tools. All analyses were conducted using the TriNetX platform’s analytical tools. These tools enabled processing of large datasets and provided robust statistical evaluations and visualizations to support epidemiological analysis.

Ethical Approval. Our study approval was obtained from the Institutional Review Board (IRB) at Virginia Commonwealth University (Approval number HM20030938), classified as a non-human subject submission to ensure adherence to ethical guidelines and patient confidentiality. Access to the TriNetX database was secured through the observational informatics program at Virginia Commonwealth University’s C. Kenneth and Dianne Wright Center for Clinical and Translational Research, following strict data governance protocols to protect patient privacy and comply with regulatory standards.

## 3. Results

Demographic Characteristics. A total of 11,400 breast cancer survivors were initially identified from the TriNetX database. Of these, 10,178 had complete outcome data regarding fall-related injuries and were used to estimate the overall prevalence of falls in the broader population. Among this group, 9.2% (N = 940) experienced fall-related injuries at any point during the study period. For the primary analysis, we applied additional inclusion criteria: female patients with Stage I–III breast cancer, identified as either Black or White, who received one or more of the specified chemotherapy agents (Doxorubicin, Cyclophosphamide, Paclitaxel, Docetaxel, or Carboplatin), and who had complete data on race and fall outcomes. This resulted in a final analytic sample of 3223 patients. All logistic regression models were conducted on this final sample. Among the 3223 breast cancer survivors included in the final analysis, all had complete data on race, chemotherapy exposure, and fall-related injuries. Of these, 1795 (55.7%) were White and 1428 (44.3%) were Black or African American. Their ages ranged from 18 to 89 years, with an average age of 69 years (±12 years). Ethnicity data was incomplete for a large proportion of the initial dataset (64.03%, N = 6512), and patients with missing race or ethnicity data were excluded from all comparative analyses. As a result, subgroup comparisons were limited to Black or African American and White patients with complete data. Therefore, subgroup comparisons were limited to Black or African American and White patients with complete data.

Racial Distribution of Breast Cancer Survivors Who Received Chemotherapy. Out of all breast cancer survivors, 28.3% (N = 3223) received chemotherapy. Among them, 55.7% (N = 1795) were White, and 44.3% (N = 1428) were Black or African American. For White breast cancer survivors, Cyclophosphamide was the most frequently used chemotherapy drug (24.9%, N = 447), followed by Paclitaxel (Taxol) (23.9%, N = 429). Docetaxel (Taxotere) was used by 19.0% (N = 341), while Carboplatin and Doxorubicin were each given to 16.1% (N = 289). A similar trend was observed among Black breast cancer survivors. Cyclophosphamide was the most commonly prescribed drug (25.2%, N = 359), followed by Paclitaxel (Taxol) (23.9%, N = 341). Doxorubicin was administered to 17.8% (N = 254), while Docetaxel (Taxotere) and Carboplatin were each given to 16.6% (N = 237). Across all patients who received chemotherapy, Cyclophosphamide was the most frequently used drug (25.0%, N = 806), followed by Paclitaxel (Taxol) (23.9%, N = 770) (Table 3).

Racial Distribution of Breast Cancer Survivors Who Received Chemotherapy and Experienced Falls. Among the 10,178 breast cancer survivors in this study, 9.2% (N = 940) experienced fall-related injuries. Of these, 41.5% (N = 390) were linked specifically to chemotherapy treatment. The likelihood of falls varies depending on the type of chemotherapy received.

For patients treated with Doxorubicin, falls were evenly distributed, with 50% (N = 30) occurring in White patients and 50% (N = 30) in Black or African American patients. Cyclophosphamide was associated with falls in 44.44% (N = 40) of White patients and 55.56% (N = 50) of Black or African American patients. Paclitaxel (Taxol) also showed an equal fall distribution, with 50% (N = 50) of falls occurring in White patients and 50% (N = 50) in Black or African American patients. For Docetaxel (Taxotere), 42.86% (N = 30) of falls occurred in White patients, while 57.14% (N = 40) were in Black or African American patients. A similar trend was observed with Carboplatin, where 42.86% (N = 30) of falls occurred in White patients, compared to 57.14% (N = 40) in Black or African American patients (Table 4).

**Figure 1:**
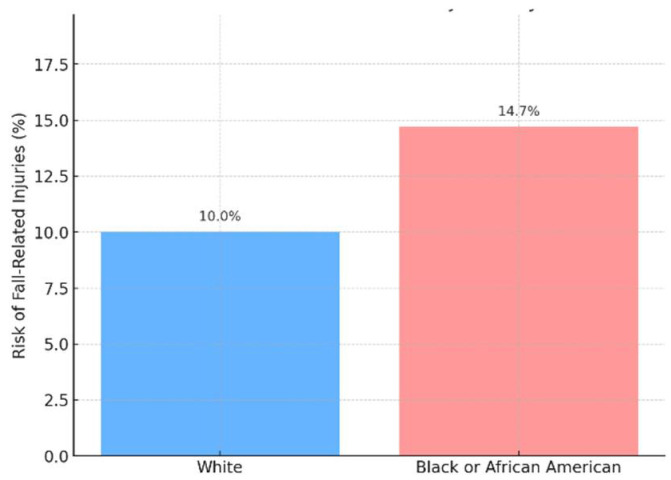
Falls risk among breast cancer survivors who received chemotherapy.

Among the 390 breast cancer survivors who received chemotherapy and experienced falls, the risk varied between White and Black or African American patients. White breast cancer survivors had a fall risk of 10.0% (N = 180), while Black or African American survivors had a higher fall risk of 14.7% (N = 210). This indicates that Black breast cancer survivors were significantly more likely to experience fall-related injuries compared to their White counterparts. The odds ratio (OR) for experiencing a fall-related injury was 0.65, indicating that White breast cancer survivors were 35% less likely to experience falls compared to their Black or African American counterparts. This difference in fall risk was statistically significant, with a *p*-value of 0.000056 (*p* < 0.001), suggesting a meaningful disparity between the two racial groups (Figure 1).

The risk of fall-related injuries among breast cancer survivors varied depending on the type of chemotherapy received and racial background. Among patients treated with Doxorubicin, the fall risk was 10.4% for White patients and 11.8% for Black patients, with an odds ratio of 0.86 (*p* = 0.681), indicating no significant difference between the two groups. A similar trend was observed for Paclitaxel (Taxol), where the fall risk was 11.7% for White patients and 14.7% for Black patients, with an odds ratio of 0.77 (*p* = 0.236), also showing no statistically significant difference.

However, for other chemotherapy agents, fall risk differed significantly by race. Among those treated with Cyclophosphamide, White patients had a fall risk of 8.9%, compared to 13.9% among Black patients (odds ratio 0.61, *p* = 0.032), indicating a higher likelihood of falls among Black survivors. A similar pattern was seen with Docetaxel (Taxotere), where 8.8% of White patients experienced falls compared to 16.9% of Black patients (odds ratio 0.48, *p* = 0.004). Likewise, for Carboplatin, the fall risk was 10.4% for White patients and 16.9% for Black patients (odds ratio 0.57, *p* = 0.038), again reflecting a significant difference (Table 5).

## 4. Discussion

This study investigates the risk of fall-related injuries among breast cancer survivors undergoing chemotherapy, with a particular emphasis on racial disparities between Black and White patients. Utilizing a retrospective cohort of 11,400 female breast cancer survivors (Stage 1–3), aged 18 and older, treated between 1 January 2019 and 31 December 2023, the study evaluates the fall risks associated with chemotherapy agents like Doxorubicin, Cyclophosphamide, Paclitaxel, Docetaxel, and Carboplatin.

Our study found that Black breast cancer survivors experienced a higher incidence of falls, particularly when treated with Cyclophosphamide, Docetaxel, and Carboplatin. However, falls are multifactorial events, influenced by factors beyond chemotherapy exposure, such as age, preexisting conditions, history of falls, mobility limitations, and access to rehabilitative care. While our analysis focuses on the association between chemotherapy types and fall risk, we acknowledge that fall-related injuries are influenced by many unmeasured factors, including comorbidities, physical functioning, neurological status, and rehabilitation access. Our findings should be interpreted as observational associations rather than evidence of causality.

This aligns with prior research indicating that chemotherapy-induced peripheral neuropathy (CIPN) is a significant risk factor for falls, especially among patients undergoing aggressive chemotherapy regimens [25]. Black patients receiving these chemotherapy agents were found to be at a significantly higher risk of falls compared to their White counterparts, suggesting potential disparities in treatment outcomes. However, these differences may also stem from unmeasured factors such as differential side effect management, provider decision-making, and access to supportive care, all of which could contribute to the higher fall risk observed among Black patients [26,27].

Mechanistically, neurotoxic agents such as taxanes (e.g., Paclitaxel, Docetaxel) and platinum compounds (e.g., Carboplatin) are known to cause sensory-motor neuropathies through multiple pathways. These include direct damage to peripheral nerve axons, inflammation of myelinated fibers, and oxidative stress targeting dorsal root ganglia. Such biological disruptions impair proprioception and motor coordination, which are essential for maintaining balance and mobility [28].

Although both Paclitaxel and Docetaxel are classified as taxanes and are known to cause chemotherapy-induced peripheral neuropathy, differences in administration schedules, cumulative dosing, and toxicity management may influence their respective impacts on fall risk. Paclitaxel is often delivered on a weekly basis at lower doses, while Docetaxel is commonly administered every three weeks at a higher dose. These variations could result in different toxicity profiles and might partially explain the observed differences in fall risk disparities between racial groups. Additionally, unmeasured factors such as comorbidities, access to supportive care, provider prescribing patterns, and patient adherence may further contribute to the differences seen in fall risk for these agents. Future studies should explore these factors to better understand regimen-specific and population-level differences in fall outcomes.

In addition to peripheral neuropathy, other well-established contributors to fall risk in cancer survivors include dizziness, anemia, fatigue, and a prior history of falls. These conditions are common in the post-treatment period and may be exacerbated by chemotherapy, comorbidities, or functional decline. Unfortunately, our dataset did not capture these variables, but their relevance is supported by prior studies and should be a focus of future research.

Additionally, prior studies suggest that socioeconomic factors may influence chemotherapy selection, with lower-cost but potentially higher-risk medications being more frequently prescribed to minority populations [29]. However, our study did not directly assess individual socioeconomic factors, such as income level, insurance status, or access to healthcare. While our results highlight racial differences in fall-related injuries following chemotherapy, further research is needed to evaluate the role of socioeconomic disparities in treatment selection and fall risk. Expanding access to equitable healthcare policies, improving insurance coverage, and ensuring better access to rehabilitative and supportive care services may help reduce disparities in chemotherapy-related fall risks among minority populations. Future interventions should focus on addressing financial barriers and developing targeted fall prevention strategies tailored to at-risk breast cancer survivors.

While socioeconomic status likely plays a role in healthcare access and treatment decisions, our dataset did not include direct Socio-Economic Status (SES) indicators (e.g., income, education, or insurance type). Thus, any references to SES in our discussion are speculative and intended to highlight areas for future research. The financial burden of chemotherapy, particularly with drugs like Cyclophosphamide, Paclitaxel, Docetaxel, and Carboplatin, significantly influences treatment decisions and outcomes for breast cancer survivors [30]. These drugs vary in cost, with Docetaxel and Carboplatin being more expensive than Cyclophosphamide. However, cost plays a crucial role in physicians’ prescribing patterns, especially when treating patients from underinsured or uninsured backgrounds [31]. In our study, Cyclophosphamide was prescribed at similar rates for both racial groups, with 24.9% (N = 447) of White patients and 25.2% (N = 359) of Black patients receiving this drug (Table 3). While prior research suggests that Black breast cancer survivors may be disproportionately affected by financial toxicity due to inadequate health insurance coverage [32]. Our findings indicate that Cyclophosphamide was not prescribed more frequently to Black patients compared to White patients.

Moreover, limited insurance coverage can delay access to high-quality supportive care, potentially exacerbating chemotherapy-related side effects and increasing the likelihood of poor outcomes. The higher costs of Docetaxel and Carboplatin, coupled with financial barriers, may make these treatments less accessible to some populations, even though they could offer more favorable side effect profiles. These findings underscore the important role that healthcare policies and insurance coverage play in ensuring equitable access to cancer treatment. Addressing these disparities requires policies that promote comprehensive care and equal access to all chemotherapy options, regardless of a patient’s financial status or race.

Several evidence-based strategies may help reduce fall risk among breast cancer survivors following chemotherapy. These include patient education on fall prevention, balance and strength training, vestibular rehabilitation programs, and home safety assessments. Incorporating such interventions into survivorship care plans may improve post-treatment safety and quality of life.

### 4.1. Strengths

The study boasts several strengths that enhance the robustness and relevance of its findings. First, the large sample size of breast cancer survivors allows for sufficient statistical power to detect meaningful differences across chemotherapy regimens and racial subgroups. This extensive dataset ensures that the results are more generalizable and reflective of broader patterns in real-world clinical settings. Additionally, the use of standardized ICD-10 codes for identifying both breast cancer diagnoses and fall-related injuries improves the accuracy and consistency of the data, minimizing potential errors in diagnosis classification and treatment outcomes. The study’s focus on multiple chemotherapy agents, including Doxorubicin, Cyclophosphamide, Paclitaxel, Docetaxel, and Carboplatin, offers a comprehensive evaluation of fall risks across different treatment modalities, providing valuable insights into drug-specific side effects that could inform future clinical guidelines. Moreover, the inclusion of racial disparities as a key component of the analysis is a significant strength, as it highlights critical healthcare inequities that affect treatment outcomes for underrepresented populations, particularly Black breast cancer survivors.

### 4.2. Limitations

Our retrospective cohort study relies on previously collected electronic health record (EHR) data, which may contain inherent limitations such as coding inaccuracies, documentation variability, and the absence of certain clinical details. The lack of longitudinal follow-up within the dataset limits the ability to validate fall-related injury reports and chemotherapy timing.

Falls are multifactorial events influenced by a range of factors beyond chemotherapy exposure, including age, prior fall history, and preexisting clinical conditions such as vestibular dysfunction, neurological and osteoarticular disorders, and visual impairment. Unfortunately, these variables were not captured in the current dataset and could not be adjusted for in our analyses. Additionally, while socioeconomic factors such as income, insurance coverage, and healthcare access are important determinants of health outcomes, TriNetX does not systematically collect these indicators. Unlike public health datasets, which often include detailed socioeconomic status (SES) measures, clinical trial and real-world evidence platforms like TriNetX primarily focus on treatment exposures and basic demographic variables, limiting our ability to assess the influence of SES on fall risk.

Another important limitation is the lack of detailed oncological staging information. While our inclusion criteria limited the study to Stage I–III breast cancer survivors, we could not stratify by specific stage within racial groups (e.g., comparing Stage I Black vs. Stage I White patients). This limits our ability to assess whether disparities in fall-related injuries were influenced by more advanced disease severity among certain subgroups. Higher-stage disease may correspond to increased frailty, mobility impairment, or more aggressive treatment regimens—all of which are relevant risk factors for falls. Future research should incorporate staging data to clarify these relationships.

Detailed age-group stratification was not performed, as the study’s primary objective focused on racial disparities in fall outcomes. Additionally, younger age groups were underrepresented, limiting the interpretability of age-based subgroup analysis.

Furthermore, the reliance on de-identified aggregate data restricts individual-level analysis, preventing adjustments for potential confounding variables such as comorbidities and concurrent medication use. While TriNetX aggregates data from multiple healthcare organizations, our study’s single-institution dataset from Virginia Commonwealth University Health Care Organization (VCU HCO) may impact generalizability. Future research incorporating broader datasets, prospective study designs, and more granular sociodemographic data would provide a more comprehensive understanding of racial disparities in fall-related injuries among breast cancer survivors. Additionally, our dataset did not include data on functional status, neurological exams, environmental hazards, or time spent in rehabilitation, all of which are key elements for a comprehensive falls risk assessment based on Evidence-Based Medicine (EBM) guidelines. This limits the clinical depth of our analysis, and our results should be viewed as hypothesis-generating rather than definitive.

## 5. Conclusions

This study identified a higher incidence of fall-related injuries among Black breast cancer survivors compared to White survivors, particularly following specific chemotherapy regimens. While falls are multifactorial events influenced by factors such as comorbidities, physical functioning, and rehabilitation access, our analysis focused on observed associations using real-world clinical data from a large, racially diverse patient population. The use of standardized inclusion criteria, complete data on race, treatment, and fall outcomes, and a defined post chemotherapy observation window strengthens the internal consistency of our findings.

We do not claim causality. Rather, we highlight important disparities that warrant attention. These preliminary results underscore the need for more comprehensive prospective research that incorporates functional status, neurological assessments, and social determinants of health. The patterns identified here offer valuable insights that can inform future studies and clinical strategies aimed at reducing inequities in cancer survivorship outcomes.

Efforts to ensure equitable treatment and supportive care throughout the cancer continuum remain critical. As healthcare systems increasingly rely on real-world evidence to guide policy and practice, identifying early signals of disparity, such as those reported here, is essential to improving quality and safety for underserved populations.

## Figures and Tables

**Table 1:** ICD-10 codes for breast cancer diagnoses and fall-related injuries used in the study.

Category	Code	Description
Breast Cancer (ICD-10)	Z85.3	Personal history of malignant neoplasm of the breast
Fall-Related Injuries (ICD-10)	W00-W19	Slipping, tripping, stumbling, and falls
	W01	Fall on same level from slipping, tripping, and stumbling
	W06	Fall from bed
	W07	Fall from chair
	W08	Fall from other furniture
	W18	Other fall on same level

**Table 2:** CPT codes for chemotherapy drugs administered to breast cancer survivors.

Drug	CPT Code	Description
Doxorubicin (Adriamycin)	J9000	Doxorubicin hydrochloride, 10 mg
Cyclophosphamide (Cytoxan)	J9070	Cyclophosphamide, 100 mg
Paclitaxel (Taxol)	J9265	Paclitaxel, 30 mg
Docetaxel (Taxotere)	J9171	Docetaxel, 1 mg
Carboplatin	J9045	Carboplatin, 50 mg

**Table 3:** Racial distribution and chemotherapy utilization among breast cancer survivors.

Chemotherapy	White Patients (N = 1795)	Black Patients (N = 1428)	Total Patients (N = 3223)
Docetaxel (Taxotere)	341 (19.0%)	237 (16.6%)	578 (17.9%)
Carboplatin	289 (16.1%)	237 (16.6%)	526 (16.3%)
Doxorubicin	289 (16.1%)	254 (17.8%)	543 (16.9%)
Cyclophosphamide	447 (24.9%)	359 (25.2%)	806 (25.0%)
Paclitaxel (Taxol)	429 (23.9%)	341 (23.9%)	770 (23.9%)
**Total**	**1795 (55.7%)**	**1428 (44.3%)**	**3223 (100%)**

**Table 4:** Racial distribution of breast cancer survivors who received chemotherapy and experienced falls.

Chemotherapy Types	Total N	White N (%)	Black or African American N (%)
Doxorubicin	60	30 (50.00%)	30 (50.00%)
Cyclophosphamide	90	40 (44.40%)	50 (55.56%)
Paclitaxel (Taxol)	100	50 (50.00%)	50 (50.00%)
Docetaxel (Taxotere)	70	30 (42.86%)	40 (57.14%)
Carboplatin	70	30 (42.85%)	40 (57.14%)

**Table 5:** Risk of fall-related injuries by race and chemotherapy type.

Chemotherapy	Race	N	% (Risk)	Odds Ratio (OR)	95% Confidence Interval (CI)	*p*-Value
Overall Risk	White	180	10.0%	Reference	-	-
	Black or African American	210	14.7%	0.65	(0.52–0.80)	0.000056
Doxorubicin	White	30	10.4%	Reference	-	-
	Black or African American	30	11.8%	0.86	(0.50–1.47)	0.681
Paclitaxel (Taxol)	White	50	11.7%	Reference	-	-
	Black or African American	50	14.7%	0.77	(0.51–1.17)	0.236
Cyclophosphamide	White	40	8.9%	Reference	-	-
	Black or African American	50	13.9%	0.61	(0.39–0.95)	0.032
Docetaxel (Taxotere)	White	30	8.8%	Reference	-	-
	Black or African American	40	16.9%	0.48	(0.29–0.79)	0.004
Carboplatin	White	30	10.4%	Reference	-	-
	Black or African American	40	16.9%	0.57	(0.34–0.95)	0.038

## Data Availability

The datasets generated and/or analyzed during the current study were extracted from the TriNetX database and are available upon request from the corresponding author due to privacy and ethical restrictions.
